# LC-MS Data set on the Malayan Deer (*Cervus timorensis*) Antler Velvet and its antibiofilm activity against *Candida* species: LC-MS Data set on the Malayan Deer (*Cervus timorensis*) Antler Velvet and its antibiofilm properties against *Candida* species.

**DOI:** 10.1016/j.dib.2021.106769

**Published:** 2021-01-16

**Authors:** Mohd Hafiz Arzmi, Akbar John, Nurul Alia Risma Rismayuddin, Norzaiti Mohd Kenali, Deny Susanti Darnis

**Affiliations:** a Department of Fundamental Dental & Medical Sciences, Kulliyyah of Dentistry, International Islamic University Malaysia, 25200 Kuantan, Pahang, Malaysia; b Cluster of Cancer Research Initiative IIUM (COCRII), International Islamic University Malaysia, 25200 Kuantan, Pahang, Malaysia; c Department of Biotechnology, Kulliyyah of Science, International Islamic University Malaysia, 25200 Kuantan, Pahang, Malaysia; d Department of Paediatric Dentistry & Dental Public Health, Kulliyyah of Dentistry, International Islamic University Malaysia, 25200 Kuantan, Pahang, Malaysia; e Department of Chemistry, Kulliyyah of Science, International Islamic University Malaysia, 25200 Kuantan, Pahang, Malaysia

**Keywords:** DAV, Oral treatment, Candida, Deer Antler, Malayan deer, *Cervus timorensis*

## Abstract

Deer antler velvet (DAV) has been traditionally used in Chinese medicine, including treatment on toothache [1]. Due to its rapid and regenerative capacity, deer antlers were proposed to be the good model for bone remodelling in mammals [2]. The data presented in this work is on the liquid chromatography and mass spectrometry (LC-MS) profile and bioactive potential of Malayan deer antler velvet (DAV) on different *Candida* species that has clinical importance. Aqueous extraction of DAV samples was subjected to Liquid chromatography quadrupole time of flight mass spectrometry (LC-QTOF-MS) profiling. Reverse phase (RP) separation was used due to the process extraction using water as a solvent to separate polar compound. The data was interpreted using Profile Analysis 2.1V. The DAV samples were also tested for the effect on the biofilm formation of seven *Candida* species in a 96 well plate [3]. The biofilms were developed for 72 h in aerobic environment. Following that, the biofilms biomass was determined using crystal violet assay.

## Abstract (author highlights)

Value of the Data

• First report on the LC-MS profile of Malayan deer (*Cervus timorensis*) antler velvet.
• The data on DAV showing reduction of *Candida* biofilm enables to understand the potential of DAV to improve oral health by inhibiting *Candida* infection.
• Data will enable understanding the importance of chemical composition of DAV in bone formation.

## Specifications Table

**Table:** 

Subject	Biology
Specific subject area	Dentistry, Oral Surgery and Medicine
Type of data	Tables
How data were acquired	Data on biofilm was acquired by incubation of *Candida* species with DAV extract to produce biofilm, stained with crystal violet and assessed using Microplate reader (Tecan NanoQuant Infinite M200, CA). Secondary metabolites dataset was acquired from the LC–MS-Quadrupled time of flight mass spectrometer online database (Vion Ion Mobility QTOF MS, Waters, USA) using both positive and negative ionization mode and were assessed using Profile Analysis Software 2.1 (Bruker, Germany).
Data format	Analysed data for *Candida* species biofilm and analysed data for LCMS identified compounds of DAV are presented within this article. Summary reports for LC-MS are provided in .pdf and Raw data for LCMS identified compounds are provided in. clsx. Both data can be accessible at Mendeley repository.
Parameters for data collection	Water extract was used for LC-MS analysis and Reversed phase column was used to separate polar compound. Mass spectrometer conditions: desolvation temperature (550 °C); desolvation gas (600 L/h); Source type (ESI); source temperature (120 °C); cone gas (50 L/h); Capillary voltage (2.50 kV); MS mode (High definition); Collision energy interval (4.00–40.00 eV). The Auto MS/MS mode was used to confirm the fragment ions. The liquid chromatography mass spectrometry (LC-MS) acquisition data were processed using Profile Analysis software (2.1) to extract the mass spectral features from samples raw data. Mono-species *Candida* biofilm reduction test was conducted in 96-well plate with nine replicates for each species (with and without DAV) cultured in RPMI-1640 media at 37 °C for 72 h. The media was replenished aseptically every 24 h at the same incubation condition.
Description of data collection	DAV materials were diluted with water. Sample was analyzed with LC-ESI-QTOF-MS system and processed using Profile Analysis software (version 2.1) *Candida* biofilms were grown in 96-well plate and was performed at nine replicates for each *Candida* species. The well with crystal violet-stained biofilms attached on the surface were read using an absorbance microplate reader at 620 nm (Tecan NanoQuant Infinite M200, CA)
Data source location	International Islamic University Malaysia (IIUM), Kuantan Pahang Malaysia (Latitude: 3.8425; Longitude: 103.2999)
Data accessibility	Complete dataset can be accessible at Mendeley Repository 10.17632/nc72jz7g4r.2
Related research article	Arzmi, M. H., Cirillo, N., Lenzo, J. C., Catmull, D. V., O'Brien-Simpson, N., Reynolds, E. C., Dashper, S., & McCullough, M. (2019). Monospecies and polymicrobial biofilms differentially regulate the phenotype of genotype-specific oral cancer cells. *Carcinogenesis, 40*(1), 184–193. 10.1093/carcin/bgy137

## 1 Data Description

Acquisition of data is possible from Liquid chromatography–mass spectrometry (LC–MS) analysis on Malayan deer antler velvet (DAV) water extract. Both Positive and negative ionization mode was analyzed.Raw OD values represents microbial biofilm density of different *Candida* species with or without DAV is presented in Table 1. LC-MS raw data was further scrutinized to identify different chemical compounds in DAV and its MS peak intensities is presented in Table 2.

**Table 1:** Raw OD values of the different *Candida* species in RPMI 1640. OD represent *Candida* biofilm density and was recorded at 620 nm wavelength in Microplate reader.

Species		Replicate 1	Replicate 2	Replicate 3	Replicate 4	Replicate 5	Replicate 6	Replicate 7	Replicate 8	Replicate 9	Mean	SD
CG	MONO	0.5872	0.4024	0.7382	0.5863	0.4023	0.7337	0.584	0.402	0.7308	0.574	0.144
	POLY DAV	0.3748	0.2548	0.2572	0.3723	0.2557	0.255	0.374	0.2549	0.2555	0.295	0.059
CT	MONO	0.4536	0.4284	0.4673	0.4546	0.4289	0.4689	0.4582	0.4328	0.4754	0.452	0.018
	POLY DAV	0.3047	0.1857	0.248	0.3033	0.1853	0.2475	0.3058	0.187	0.2495	0.246	0.051
CK	MONO	0.2597	0.459	0.639	0.2589	0.4571	0.634	0.2582	0.4541	0.6312	0.450	0.163
	POLY DAV	0.2725	0.2468	0.4187	0.2707	0.2374	0.4148	0.2721	0.2368	0.4169	0.310	0.082
CP	MONO	0.4112	0.3615	0.8658	0.413	0.3621	0.866	0.416	0.3645	0.8749	0.548	0.241
	POLY DAV	0.5348	0.5738	0.4972	0.5371	0.5764	0.4996	0.5404	0.5785	0.5002	0.538	0.034
CL	MONO	0.4756	0.5582	0.4184	0.472	0.5508	0.4126	0.4716	0.5499	0.4111	0.480	0.060
	POLY DAV	0.3807	0.3231	0.3052	0.3786	0.3214	0.3045	0.3801	0.3219	0.3056	0.336	0.034
CD	MONO	0.5983	0.4833	0.3347	0.6014	0.4845	0.3358	0.6109	0.4885	0.3393	0.475	0.116
	POLY DAV	0.4949	0.2333	0.3469	0.4926	0.2326	0.3453	0.4961	0.2343	0.3478	0.358	0.113
ATCC	MONO	0.1498	0.1121	0.1379	0.1489	0.1112	0.1373	0.1489	0.1112	0.1371	0.133	0.017
	POLY DAV	0.303	0.434	0.4623	0.301	0.4342	0.4595	0.302	0.4348	0.4615	0.399	0.074
ALC2	MONO	0.1064	0.2382	0.1975	0.1064	0.239	0.1974	0.1069	0.2429	0.199	0.182	0.059
	POLY DAV	0.2903	0.3473	0.2608	0.29	0.3472	0.2603	0.2925	0.35	0.2618	0.300	0.038
ALC3	MONO	0.4025	0.3486	0.6585	0.3935	0.3455	0.6491	0.3985	0.3461	0.6473	0.466	0.141
	POLY DAV	0.279	0.2667	0.2723	0.2771	0.2656	0.2706	0.2778	0.2673	0.2714	0.272	0.005

*Note: Mono represents *Candida* monoculture while poly represents *Candida* cultured with DAV supplement. CG: *C. glabrata,* CT: *C. tropicalis,* CK: *C. krusei*, CP: *C. parasilopsis,* CL: *C. lusitaniae*, CD: *C. dubliniensis,* ATCC: *C. albicans* MYA-4901, ALC2: *C. albicans* HIV isolates, ALC3: *C. albicans* oral cancer isolates.

**Table 2:** Chemical compounds identified in DAV and its MS peak intensities of the LC-QTOF-MS.

No.	Component name	Formula	Identification status	Observed neutral mass (Da)	Observed m/z	Mass error (mDa)	Mass error (ppm)	Observed RT (min)	Response	Adducts	Observed CCS (Å²)	Total Fragments Found
1	Benzopyran derivativeⅡ	C32H30O7	Identified	526.2009	525.1936	1.7	3.3	1.11	2075	-H	213.32	0
2	Meliadanoside A	C16H24O10	Identified	376.1359	421.1341	-1.0	-2.4	1.22	2374	+HCOO	195.83	0
3	Octahydrocurcumin	C21H28O6	Identified	376.1859	421.1841	-2.6	-6.3	2.12	1304	+HCOO	198.21	0
4	3′,4′-Dimethoxy- isoflavan-7,2′-di-O-β-D- glucoside	C29H38O15	Identified	626.2216	671.2198	0.5	0.8	2.38	1090	+HCOO	254.40	0
5	4- Hydroxyacetophenone	C8H8O2	Identified	136.0521	181.0503	-0.3	-1.8	2.87	1848	+HCOO , -H	144.29	1
6	Methyl-β-orsellinate	C9H10O4	Identified	182.0576	181.0503	-0.3	-1.8	2.87	1848	-H	144.29	3
7	Didemethoxylcurcumin	C19H16O4	Identified	308.1024	353.1006	-2.5	-7.0	5.08	1230	+HCOO	171.99	0
8	Moracin E	C19H16O4	Identified	308.1024	353.1006	-2.5	-7.0	5.08	1230	+HCOO	171.99	0
9	Moracin E	C19H16O4	Identified	308.1024	353.1006	-2.5	-6.9	5.51	4981	+HCOO	239.37	1
10	Didemethoxylcurcumin	C19H16O4	Identified	308.1024	353.1006	-2.5	-6.9	5.51	17118	+HCOO	196.06	3
11	Apocynin	C9H10O3	Identified	166.0627	165.0554	-0.3	-1.9	5.51	1099	-H	201.32	2
12	2′-Hydroxy-3′,4′- dimethoxy-isoflavan-7- O-β-D-glucoside	C23H28O10	Identified	464.1635	509.1617	-4.7	-9.3	16.49	1066	+HCOO	224.36	0
13	Feralolide	C18H16O7	Identified	344.0886	343.0813	-1.0	-2.9	16.69	2460	-H	179.09	1
14	(3R,4R)-3,4-trans-7,2′- Dihydroxy-4′- methoxy-4-[(3R)-2′,7- dihydroxy-4′-methoxy- isoflavan-5′-yl]-isoflavan	C32H30O8	Identified	542.1936	587.1918	-0.5	-0.9	18.52	1177	+HCOO , -H	242.15	1
15	Dichotomitin	C18H14O8	Identified	358.0708	381.06	2	5.1	0.81	2004	+Na, +K	183.78	1
16	6-Aldehydo-7-methoxy- isoophiopogonone A	C20H16O7	Identified	368.0919	391.0811	2.3	5.9	0.97	2090	+Na	181.97	2
17	Dichotomitin	C18H14O8	Identified	358.071	381.0603	2.2	5.7	0.98	1279	+Na	184.29	0
18	Bavachinin	C21H22O4	Identified	338.1506	361.1398	-1.2	-3.3	1.81	1161	+Na	181.66	0
19	Mirificin	C26H28O13	Identified	548.1485	549.1557	-4.5	-8.3	2.02	2036	+H, +Na	258.43	0
20	Naringenin-4′- glucoside-7-rutinoside	C33H42O19	Identified	742.2294	765.2186	-2.7	-3.5	2.02	1049	+Na	260.68	1
21	5-Hydroxyauranetin	C20H20O8	Identified	388.1159	395.1313	0.1	0.2	2.54	1504	+Li	194.15	0
22	(-)-Epiafzelechin-3-O- (6"-O-acetyl)-β-D- allosepyranoside	C23H26O11	Identified	478.1467	479.1539	-0.9	-1.8	5.07	3463	+H	225.46	1
23	Silymonin	C25H22O9	Identified	466.122	473.1375	-4.3	-9.2	5.07	2073	+Li	224.21	0
24	Daidzin_1	C21H20O9	Identified	416.1114	439.1006	0.7	1.6	5.33	1112	+Na	196.3	0
25	3,4,2′-Trihydroxychal- cone-4′-O-β-D- glucopyranoside	C21H22O10	Identified	434.1225	457.1118	1.2	2.7	5.33	1272	+Na	199.28	0
26	Pinnatifinoside B	C23H20O10	Identified	456.1045	457.1118	-1.2	-2.5	5.33	1272	+H	199.28	1
27	Bavachalcone	C20H20O4	Identified	324.1343	347.1235	-1.9	-5.5	6.01	4354	+Na	182.22	0
28	Corylifolinin	C20H20O4	Identified	324.1343	347.1235	-1.9	-5.5	6.01	4354	+Na	182.22	0
29	Hibiscetin-3-O- glucoside	C21H20O14	Identified	496.0851	497.0923	-0.2	-0.5	7.47	6132	+H	217.13	0
30	Norcimifugin	C15H16O6	Identified	292.094	299.1094	-0.7	-2.4	11.86	1429	+Li	167.93	0
31	5-Hydroxy-7,8- dimethoxy-6-methyl-3- (3′,4′-dihydroxybenzyl) chroman-4-one	C19H20O7	Identified	360.1207	367.1362	-0.2	-0.5	14.98	1037	+Li	177.93	0
32	Sophoranodichromane D	C25H28O5	Identified	408.1961	415.2115	2.4	5.7	16.67	12569	+Li, +H	205.99	17
33	(2S)-3′,4′- Methylenedioxy-5,7- dimethoxyflavane	C18H18O5	Identified	314.1151	337.1043	-0.3	-0.9	16.7	100608	+Na, +Li	179.5	5
34	Ophiopogonanone B	C18H18O5	Identified	314.1151	337.1043	-0.3	-0.9	16.7	100608	+Na, +Li	179.5	6
35	Kuwanon S	C25H26O5	Identified	406.1777	407.185	-0.3	-0.8	16.71	1296	+H, +Li, +Na	209.9	42
36	Neokurarinol	C27H34O7	Identified	470.2297	471.237	-0.8	-1.6	16.72	1267	+H	219.52	31
37	Glabrol	C25H28O4	Identified	392.1994	393.2067	0.6	1.6	16.72	2413	+H, +Li	195.67	34
38	3-(4′-Hydroxy- benzyl)-5,7- dihydroxy-6,8-dimethyl- chroman-4-one	C18H18O5	Identified	314.1147	337.1039	-0.8	-2.3	16.95	10015	+Na	178.84	0
39	(2S)-3′,4′- Methylenedioxy-5,7- dimethoxyflavane	C18H18O5	Identified	314.115	337.1043	-0.4	-1.2	17.28	15479	+Na	179.31	0
40	3-(4′-Hydroxy- benzyl)-5,7- dihydroxy-6,8-dimethyl- chroman-4-one	C18H18O5	Identified	314.115	337.1043	-0.4	-1.2	17.28	15479	+Na	179.31	0
41	(2S)-3′,4′- Methylenedioxy-5,7- dimethoxyflavane	C18H18O5	Identified	314.1153	337.1045	-0.1	-0.3	18.03	12092	+Na	179.64	0
42	3-(4′-Hydroxy- benzyl)-5,7- dihydroxy-6,8-dimethyl- chroman-4-one	C18H18O5	Identified	314.1153	337.1045	-0.1	-0.3	18.03	12092	+Na	179.64	0
43	3-(4′-Hydroxy- benzyl)-5,7- dihydroxy-6,8-dimethyl- chroman-4-one	C18H18O5	Identified	314.1173	321.1327	1.8	5.7	18.46	1800	+Li	181.35	1
44	3-(4′-Hydroxy- benzyl)-5,7- dihydroxy-6,8-dimethyl- chroman-4-one	C18H18O5	Identified	314.1154	321.1309	0	0	18.6	1458	+Li	178.4	0

## 2 Experimental Design, Materials and Methods

### 2.1 DAV collection and sample preparation

Male Malayan deer antler was collected from local company; D'Paradise Deer Valley and Exotic Farm Sdn. Bhd. Dried samples was powdered using variable speed laboratory blender (Waring®, USA). 10 g of powdered sample was soaked in 450 ml double distilled deionized water with periodic shaking for 30 min intervals for 8 h; then the mixture will be allowed to settle for 16 h. After filtration, the residue was washed with a small portion of water and followed by freeze-drying (Martin Christ, Germany).

### 2.2 Mono biofilm reduction test

A total of 7 Candida species (*C. glabrata* ATCC 90030*, C. tropicalis* ATCC 13803*, C. krusei* ATCC 14243*, C. parasilopsis* ATCC 22019*, C. lusitaniae* ATCC 64125*, C. dubliniensis* ATCC MYA-2975*, C. albicans* ATCC MYA-4901) were procured from American Type Culture Collection (ATCC). While ALC2 (isolate from HIV positive patient) and ALC3 (isolate from Oral cancer patient) representing *Candida albicans* isolates from Melbourne dental school, University of Melbourne, Australia were tested for biofilm formation with or without DAV extract of known concentration in RPMI-1640 media following the previously established procedure [3], [4]. Nine replicates for each species were accessed in 96 well-plate. Samples were incubated at 37 °C for 72 h and the fungal density was determined using Microplate reader (Tecan NanoQuant Infinite M200, CA) at 620 nm wavelength (Table 1). All chemicals and reagents used were of analytical grade procured from Thermo Scientific.

### 2.3 LC-MS analysis

The DAV solution was prepared for LC–MS analysis by diluting the extract in aqueous solution. Samples were flow through on Column ACQUITY UPLC-BEH C18, 2.1 mm x 100 mm (Waters, USA) at the rate of 0.5 ml/min. The mobile phase was prepared using the binary solvent manager with solvent A and B.  Briefly, the gradients elution were 99% A and 1% B (0.00 min) 99% A and 1% B (0.00–0.50 min), 65% A and 35% B (0.50–16.00 min), 0% A and 100% B (16.00–18.00 min), 99% A and 1% B (18.00–20.00 min), with the flow rate of 0.6 mL/min. The solvent A is made of 0.1% formic acid (Sigma Aldrichs, Germany) plus water (Milli-Q grade, v/v). However, solvent B was acetonitrile at a seal wash time and highest-pressure limit of 5 min and 1800 psi, respectively. Positive and negative ionization mode was performed at the following setting: capillary voltage: 2.50 kV, nebulizer pressure: 1.2 bar, drying gas: 8 l/min at 200 °C, mass range: 50–1000 m/z [5]. The sample was analyzed using LC-ESI-QTOF-MS (Vion Ion Mobility QTOF MS, Waters, USA) and processed using Profile Analysis 2.1 (Bruker, Germany).

## Ethics Statement

This research work does not require ethical approval.

## CRediT Author Statement

**Arzmi M.H:** Conceptualization, Methodolog;, **John B.A.:** Data interpretation, Writing - manuscript; **Rismayuddin N.A.R, Kenali N.M:** Laboratory analysis; **Arzmi M.H:** Supervision; **Darnis D.S:** Validation; **Arzmi M.H., John B.A., Rismayuddin N.A.R.:** Writing - Reviewing and Editing. All authors equally contributed to this manuscript.

## Declaration of Competing Interest

Authors have no competing interest.
